# Contributions to Obstetric Jurisprudence

**Published:** 1859-03

**Authors:** Horatio R. Storer

**Affiliations:** Boston


ART. I.—*Contributions to Obstetric Jurisprudence.* By HORATIO R. STORER, M.D., of Boston.

### CRIMINAL ABORTION (CONTINUED.)

### II. ITS FREQUENCY, AND THE CAUSES THEREOF.

THOUGH we cannot at once, and in exact figures, show the yearly amount of criminal abortion in this country, statistics on the subject being necessarily imperfect or wanting, we may yet arrive at an approximate result. This is done by an easy and reliable process of induction, the several factors of which, each of itself rendering probable the conclusion, tend, when combined, to make it almost absolutely certain.

We are to consider, in this connection, the evidence afforded by—

I.—The comparative increase of population.

II.—The published records of still-births.

III.—The number of arrests, or trials for abortion.

IV.—The published number of immediate maternal deaths.

V.—The pecuniary success of abortionists, and abortion-producing nostrums.

VI.—The comparative size of families in present and past times.

VII.—The experience of physicians—direct from applications for abortion and actual cases, and indirect from their results.

Several of these points are as yet almost wholly uninvestigated. They are stated, therefore, with care, as bearing decidedly on the question at issue, and as tending to provoke still further research.

To go into an elaborate comparison of our national and state censuses with themselves, past and present, with each other, and with those of similar communities abroad, involving, as it would do, intricate calculations regarding the effect of emigration from state to state, and from nation to nation, the increase of urban population, and the frequent decrease of rural, is not our present intention, nor is it necessary. By considering this point in connection with that immediately succeeding it, with which it is intimately related, its bearing and importance will at once be seen.

Statistics in this country are as yet so imperfect, that we are necessitated to a process of deduction. If it can be shown that a state of things prevails elsewhere to a certain extent, explainable only on one supposition, and that the same state of things prevails in this country to a greater extent, all other causes, save the one referred to, being in great measure absent, little doubt can be entertained of the part this plays; but if it can, in addition, be proved that this cause must necessarily be stronger with us than elsewhere, then its existence becomes morally certain. Accordingly, if we find that in another country living births are steadily lessening in proportion alike to the population and to its increase, that natural or preventive causes are insufficient to account for this, while the proportion of still-births and of known abortions is constantly increasing, and these last bear an evident yet increasing ratio to the still-births; that in this country the decrease of living births, and the increase of still-births, are in much greater ratio to the population, and the proportion of premature births is also increasing; that these relations are constant and yearly more marked, we are justified in supposing that abortions are at least as frequent with us, and probably more so.

In many countries of Europe, it has been ascertained that the "fecundity" of the population, or the rate of its annual increase, is rapidly diminishing.*

* Many of the statistics now presented we have also embodied in a paper upon the decrease of the rate of increase of population now obtaining in Europe and America, read before the American Academy of Arts and Sciences, December 14th, 1858, as a contribution to the Science of Political Economy.

In Sweden, it has lessened by one-ninth in 61 years. In Prussia, by a third in 132 years. In Denmark, by a quarter in 82 years. In England, by two-sevenths in a century. In Russia, by an eighth in 28 years. In Spain, by a sixth in 30 years. In Germany, by a thirteenth in 17 years. In France, by a third in 71 years.*

Or, in other words:

In Sweden it has lessened by a fifth; in Prussia, by a fourth; in Denmark and England, by a third; and in Russia, Spain, Germany, and France by a half, in a single century.

For the sake of convenience, larger bodies of statistics existing concerning it, and from the fact that it represents the extreme of the alleged decrease, we take France for our comparisons.

In France at large, according to the official returns as analyzed by Legoyt, the increase of the population which, from 1801 to 1806, was at the rate of 1·28 per cent. annually, from 1806 to 1846 had fallen to about ·5 per cent.† The exact ratio of decrease after this point is better shown by the figures themselves. The increase from 1841 to 1846 was 1,200,000; from 1846 to 1851, 380,000; from 1851 to 1856, 256,000.

In England, during this latter period, with a population of but one-half the size, the returns of the Registrar-General show a relative increase nine times greater.‡ In thirty-seven years, from 1817 to 1854, the mean annual increase in France was not more than 155,929, yet in five years, from 1846 to 1851, it had fallen to 76,000 yearly, and from 1851 to 1856, to 51,200, and this with a population ranging from twenty-nine to thirty-four millions.

A comparison of these facts, with those obtaining in other European States, will make the above still more evident. We now quote from Rau.§

| | Rate of Increase. Per Cent. | | Rate of Increase. Per Cent. |
|---|---|---|---|
| Hungary, according to Rohrer | 2·40 | Saxony, from 1815 to 1830 | 1·15 |
| England, from 1811 to 1821 | 1·78 | Baden (Heunisch,) from 1820 to 1830 | 1·13 |
| " from 1821 to 1831 | 1·60 | Bavaria, from 1814 to 1828 | 1·08 |
| Prussia, from 1816 to 1827 | 1·54 | Naples, from 1814 to 1824 | 0·83 |
| " from 1820 to 1830 | 1·37 | France (Mathieu,) from 1817 to 1827 | 0·63 |
| " from 1821 to 1831 | 1·27 | France, more recently, (De Jonnés) | 0·55 |
| Austria, (Rohrer) | 1·30 | | |
| Scotland, from 1821 to 1831 | 1·30 | | |
| Netherlands, from 1821 to 1828 | 1·28 | | |

* MOREAU DE JONNÉS, Eléments de Statistique, 1856, p. 202.

† Journal des Economistes, March and May, 1847.

‡ Edinburgh Review, Jan. 1857, p. 342. Med. Times and Gazette, May, 1857, p. 462.

§ Lehrbuch der Politischen Oekonomie.

A similar and corroborative table, containing additional matter, is given by Quetelet;* its differences from the preceding are owing to its representing a series of different years.

| | Rate of Increase. Per Cent. | | Rate of Increase. Per Cent. |
|---|---|---|---|
| Ireland | 2·45 | Austria | 1·30 |
| Hungary | 2·40 | Bavaria | 1·08 |
| Spain | 1·66 | Netherlands | 0·94 |
| England | 1·65 | Naples | 0·83 |
| Rhenish Prussia | 1·33 | France | 0·63 |

And more recently, Legoyt brings up these results to the close of 1846.†
As shown by the census, the rate of increase was, in

| | Per Cent. | | Per Cent. |
|---|---|---|---|
| Great Britain, exclusive of Ireland | 1·95 | Sardinia | 1·08 |
| Prussia | 1·84 | Holland | 0·90 |
| Saxony | 1·45 | Austria | 0·85 |
| Norway | 1·36 | Sweden | 0·83 |
| | | France | 0·68 |

Or, as shown by the annual excess of births over deaths, and therefore more reliable—

| | Per Cent. | | Per Cent. |
|---|---|---|---|
| Norway | 1·30 | Austria | 0·90 |
| Prussia | 1·18 | Saxony | 0·90 |
| Sweden | 1·14 | Hanover | 0·85 |
| Holland | 1·03 | Belgium | 0·76 |
| Wurtemberg | 1·00 | Bavaria | 0·71 |
| Great Britain, exclusive of Ireland | 1·00 | Russia | 0·61 |
| Denmark | 0·95 | France | 0·50 |

In four departments of France, among which are two of the most thriving of Normandy, the deaths actually exceed the births.‡

From the above facts, it would naturally be supposed that the percentage of births to the whole population must be smaller than in other European countries, and from the lessened annual rate of increase of the population, that the proportionate number of births must be decreasing in similar ratio. This is found, indeed, to be the case.

From large statistics furnished by De Jonnés, we have compiled the following table of the comparative ratios of births to the population in the principal countries of Europe:—

* Sur l'Homme et la Developpement de ses Facultés, tome i. ch. 7.
† Journal des Economistes, May, 1847.
‡ MILL, Principles of Political Economy, i. p. 343.

| | Ratio. | | Ratio. |
|---|---|---|---|
| Venice and Dependencies, 1827 | 1 to 23 | Sweden, 1825 | 1 to 28 |
| Tuscany, 1834 | " | Holland, 1832 | " |
| Lombardy, 1828 | 1 to 24 | Austria, 1829 | " |
| Russia, 1835 | 1 to 25 | Belgium, 1836 | " |
| Wurtemberg, 1821 to 1827. | " | Bavaria, 1825 | " |
| Prussia, 1836 | " | Two Sicilies, 1831 | " |
| Mecklenburg, 1826 | 1 to 26 | Sweden and Norway, 1828 | 1 to 30 |
| Sardinia, 1820 | " | Denmark, 1833 | " |
| Naples and Dependencies, 1830 | " | Roman States, 1836 | " |
| Greece, 1828 | " | Turkey, 1835 | " |
| Poland, 1830 | 1 to 27 | Hanover, 1835 | 1 to 31 |
| Ireland, 1821 to 1831 | " | Sicily, 1832 | " |
| Germany, 1828 | " | Austria, 1828 to 1830 | 1 to 32 |
| Switzerland, 1828 | " | Great Britain, 1821 to 1831 | " |
| Spain, 1826 | " | Scotland, 1821 to 1831 | 1 to 34 |
| Portugal, 1815 to 1819 | 1 to 27·5 | England, 1821 to 1831 | 1 to 35 |
| | | Norway, 1832 | " |
| | | France, 1771 to 1851 | 1 to 25 to 1 to 37 |

In a total population, at different periods, of 232,673,000, there were 8,733,000 births; whence an average on the grand scale of one birth to every 26·6 individuals.

In France, however, the ratio has been steadily lessening, as seen by the following table :—

| | Ratio of births. | | Ratio of births. |
|---|---|---|---|
| 1771 to 1775 | 1 to 25 | 1836 to 1840 | 1 to 34 |
| 1801 to 1810 | 1 to 30 | 1841 to 1845 | 1 to 35 |
| 1811 to 1825 | 1 to 32 | 1846 to 1850 | 1 to 37 |
| 1826 to 1836 | 1 to 33 | | |

The position of France, as compared with the rest of Europe, in respect to the ratio of births to the population at different periods, is made still more manifest by another table :—

| Annual ratio of births. | |
|---|---|
| 1 to 23 | Venetian Provinces, 1827; Tuscany, 1834. |
| 1 to 23·5 | Kingdom of Naples, 1822 to 1824. |
| 1 to 24 | Tuscany, 1818; Sicily, 1824; Lombardy, 1827 to 1828; Russia, 1831. |
| 1 to 24·5 | Prussia, 1825 to 1826. |
| 1 to 25 | France, 1781; Austria, 1827; Russia, 1835; Prussia, 1836. |
| 1 to 26 | Sardinia, 1820; Hanover, Wurtemberg and Mecklenburg, 1826; Greece, 1828; Naples, 1830. |
| 1 to 27 | Spain, 1826; Germany and Switzerland, 1828; Poland, 1830; Ireland, 1831. |
| 1 to 27·5 | Portugal, 1815 to 1819. |

| | |
|---|---|
| 1 to 28 | Holland, 1813 to 1824; Bavaria and Sweden, 1825; Austria, 1829; Belgium, 1836. |
| 1 to 29 | Canton Lucerne, 1810; Holland, 1832. |
| 1 to 29·8 | France, 1801. |
| 1 to 30 | Sweden and Norway, 1828; Belgium, 1832; Denmark, 1833; Turkey, 1835; States of the Church, 1836. |
| 1 to 31 | Sicily, 1832; Hanover, 1835. |
| 1 to 31·4 | France, 1811. |
| 1 to 31·6 | France, 1821. |
| 1 to 32 | Austria, 1830; Great Britain and Switzerland, 1831. |
| 1 to 33 | France, 1828 to 1831. |
| 1 to 34 | Norway and Holstein, 1826; Scotland, 1831; France, 1834 to 1841. |
| 1 to 35 | Denmark, 1810; England, 1831; Norway, 1832. |
| 1 to 37 | France, 1851. |

In Paris, strange to say, the decrease in the ratio of births to the population, though decided and steady, has not, in actual proportion, been as great as in the empire at large, showing that the cause, whatever we find it to be, is not one depending on the influence of a metropolis alone for its existence.

From 1817 to 1831 there averaged, in Paris, one birth to 26·87 inhabitants; but from 1846 to 1851, one to 31·98.*

Again, as might have been expected, we find that the proportion of still-births, in which we must include abortions, as has hitherto been done, however improperly, in all extensive statistics, is enormous, and is steadily increasing. To show this the more plainly, we first present a table of the ratio of still-births to the living births in the various countries of Europe.†

| | | | |
|---|---|---|---|
| Geneva,‡ 1824 to 1833 | 1 to 17 | Prague, 1820 | 1 to 30 |
| Berlin Hospitals, 1758 to 1774 | 1 to 18 | London Hospitals, 1749 to 1781 | 1 to 31 |
| Paris Maternité,§ 1816 to 1835 | 1 to 20 | Vienna, 1823 | 1 to 32 |
| Sweden, 1821 to 1825 | 1 to 23·5 | Austria, 1828 | 1 to 49 |
| Denmark, 1825 to 1834 | 1 to 24 | France at large, 1853 | 1 to 24 |
| Belgium,‖ 1841 to 1843 | 1 to 24·2 | Department of Seine | 1 to 15 |
| Prussia,¶ 1820 to 1834 | 1 to 29 | Paris,** 1836 to 1844 | 1 to 14·3 |
| Iceland, 1817 to 1828 | 1 to 30 | " 1845 to 1853†† | 1 to 13·8 |

* HUSSON, Les Consummations de Paris, 1856. † Compiled from DE JONNÉS.

‡ 10,925 births; 646 still-births.

§ Births, 52,538; still-births, 2624.

‖ Compiled from QUETELET, Theory of Probabilities, p. 152; 406,073 living births; 16,767 still-births.

¶ Births, 7,593,017; still-births, 257,068. 1839–41, 1 to 26; ELLIOTT, Hunt's Merchants' Magazine, July, 1856.

** 2080 still-births. †† 2349 still-births.

The proportion of still-births in the rural districts of France is governed by the same laws as in the metropolis.

In 363 provincial towns the ratio was, from

| | |
|---|---|
| 1836 to 1845 . . . . . 1 to 19·55 | 1846 to 1850 . . . . . . 1 to 18·8 |

While districts more thinly populated gave, from

| | |
|---|---|
| 1841 to 1845 . . . . . . 1 to 29 | 1846 to 1850 . . . . . . 1 to 27* |

In Belgium, during a similar period, the ratio was much the same.†

| | |
|---|---|
| 1841 to 1843, in towns . 1 to 16·1 | 1841 to 1843, in country . 1 to 29·4 |

The apparent discrepancy between city and country, noticed as equally obtaining in Belgium and France, is chiefly owing to greater negligence of the country officials in registering the still-births, and to the fact, as we have seen in Paris, that the ratio of births to the population is greater in the city than in the country at large.

Finally, while the proportion of still-births to the whole number is greatly increasing in Paris, so is the number of known abortions.

We omit, for the present, the evidence afforded by arrests and trials, which we might here have turned to account. At the Morgue, which represents but a very small fraction of the fœtal mortality of Paris, and in this matter almost only crime, there were deposited during the eighteen years preceding 1855, a total of 1115 fœtuses,‡ of which 423 were at the full term, and 692 were less than nine months; and of these last, 519, or five-sixths, were not over six months, a large proportion of them showing decided marks of criminal abortion.

Again, of the 692 fœtuses of less than nine months, deposited at the Morgue during these eighteen years, 295 were between 1836 to 1845, an average, at that time, of 32·7 yearly; and from 1846 to 1855 there were 397, an average of 44·1. During the means of these periods the births in France were as follows§:—In 1841, 1,005,203, and in 1851, 1,037,040, from which it is evident that there was deposited at the Morgue, in 1841, one infant, dead from abortion, to every 30,700 births; and in 1851, one to every 23,500. The increased ratio is seen to be striking; it will hereafter become apparent that the increase is far greater in reality.

We turn now to our own country, to which the City of New York holds much the same relation, as respects public opinion no less than in other matters, that Paris holds to France.

Since 1805, when returns were first made to the Registry of New York, the number, proportionate as well as actual, of fœtal deaths, has steadily and rapidly increased. With a population at that time (1805) of 76,770, the number of still and premature births was 47; in 1849, with a popula-

---

* De Jonnés, loc. cit., p. 239.

† Quetelet, loc. cit., p. 152.

‡ Register of the Morgue.

§ De Jonnés, loc. cit., p. 193.

tion estimated at 450,000, the number had swelled to 1320.* Thus, while the population had increased only *six* times since 1805, the annual number of still and premature births had multiplied over *twenty-seven* times.

The following table shows the rapidity of this increase :—The ratio of fœtal deaths to the population, was in

| | | | |
|---|---|---|---|
| 1805 . . . . . . . | 1 to 1633·40 | 1830 . . . . . . . | 1 to 597·60 |
| 1810 . . . . . . . | 1 to 1025·24 | 1835 . . . . . . . | 1 to 569·88 |
| 1815 . . . . . . . | 1 to 986·46 | 1840 . . . . . . . | 1 to 516·02 |
| 1820 . . . . . . . | 1 to 654·52 | 1845 . . . . . . . | 1 to 384·68 |
| 1825 . . . . . . . | 1 to 680·68 | 1849 . . . . . . . | 1 to 340·90 |

In the three years preceding 1849, there were registered in New York 400 premature births, and 3139 children still-born; a total of 3539, representing at that time a yearly average of some 1200 fœtal deaths. While it will be shown hereafter that a large proportion of the reported premature births must always be from criminal causes, and that though almost all the still-births at the full time, even from infanticide, are necessarily registered, but a small proportion of the abortions and miscarriages occurring are ever reported to the proper authorities, it will immediately be made apparent that at the present moment the abortion statistics of New York are far above those of 1849.

In the three years preceding 1857, there were registered in New York 1196 premature, and 4735 still-births,† a total of 5931, representing a yearly average of some 2000 fœtal deaths; showing that in the short space of seven years, the number of fœtal deaths in New York, already enormous, had very nearly doubled.

Again, in 1856, the total number of births at the full time in New York, was 17,755; of these, 16,199 were living;‡ proving that of children at the full time alone, setting aside the great number of viable children born prematurely, and the innumerable earlier abortions not recorded, one in every 11·4 is born dead.

From foreign statistics on a large scale, it is found that the proportion of still-births, even allowing a wide margin for criminal causes, does not, in those countries, drop below 1 in 15, and this in France, ranging from that number up to 1 in 30 or 40 of the whole number of births reported. We have already given a table upon this point.

In Geneva, out of 10,925 births occurring from 1824 to 1833, 1221 of them being *illegitimate*, and therefore to be supposed liable to a large percentage of deaths from criminal causes, there were only 646 fœtal deaths; a proportion of 1 in 17.

* Report of City Inspector of New York for 1849.

† City Inspector's Report for 1856. ‡ Ibid.

In Belgium there were 29,574 *illegitimate* births from 1841 to 1843, and of these, 1766 were born still;* 1 in 16·8.

In New York, from 1854 to 1857, there were 48,323 births, and 5931 still-births, at the full time and prematurely; or in other words, 1 to every 8·1 was born dead.

The ratio of still-births in New York, including, as we have seen, abortions, is steadily increasing, as seen by the following table,† in which we have compared the still-births, supposable perhaps of accidental value, with the general mortality, whose value is at least as accidental, if not more so. The evidence, like that already furnished, is astounding.

| | Total mortality. | Still-births. | Ratio. |
|---|---|---|---|
| 1804 to 1809 . . . . . . . . . | 13,128 | 349 | 1 to 37·6 |
| 1809 to 1815 . . . . . . . . . | 14,011 | 533 | 1 to 26·3 |
| 1815 to 1825 . . . . . . . . . | 34,798 | 1,818 | 1 to 19·1 |
| 1825 to 1835 . . . . . . . . . | 59,347 | 3,744 | 1 to 15·8 |
| 1835 to 1855 . . . . . . . . . | 289,786 | 21,702 | 1 to 13·3 |
| 1856‡ . . . . . . . . . . . | 21,658 | 1,943 | 1 to 11·1 |

The frequency of abortions and premature births reported from the practice of physicians, and thus to a certain extent, but not entirely, likely to be of natural or accidental origin, is as follows:—

In 41,699 cases registered by Collins, Beatty, La Chapelle, Churchill, and others,§ there were 530 abortions and miscarriages. Here all the abortions were known; their proportion was 1 in 78·5.

In New York, from 1854 to 1857, there were 48,323 births at the full time reported, and 1196 premature. Here all the abortions were not known, probably but a very small fraction of them; the proportion was 1 in 40·4.

Finally, we compare the recorded premature still-births of New York, with those still at the full time.

In the seventeen years from 1838 to 1855,|| there were reported 17,237 still-births at the full time, and 2710 still prematurely; the last bearing the proportion of 1 to 6·3.

In the nine years, from 1838 to 1847, omitting 1842 for reasons stated below, there were 632 premature still-births, and 6445 still at the full time; a yearly average of 1 in 10·2.

* Compiled from QUETELET, loc. cit., p. 152.

† Compiled from City Inspector's Report for 1855.

‡ Ibid. for 1856.

§ CLAY, Obstetric Cyclopedia, p. 21.

|| Separate records of the premature births in New York were not made before this period. They were not rendered in 1842; I have therefore omitted in the calculation the still-births of the same year. For a series of the official reports, I am indebted to the present City Inspector, Mr. Geo. W. Morton.

In the eight years, from 1848 to 1855, there were 2078 premature still-births, and 10,792 still at the full time; an average of 1 in 5.

While in 1856 there were 387 still prematurely, and 1556 at the full time; or 1 in 4·02.

From these figures there can be drawn but one conclusion, that criminal abortion prevails to an enormous extent in New York, and that it is steadily and rapidly increasing. "We cannot refer," was well said by a former inspector of that city,* "such a hecatomb of human offspring to natural causes." We shall now endeavor to prove this point by other reasoning.

That our deductions concerning the population and births of France are perfectly legitimate, is admitted beforehand by the leading political economists of the day; ignorant as they were in its various relations of much of the evidence now brought forward, and of the conclusion to which the whole matter, directly and with almost mathematical exactness, is proved to tend.

"In France," remarks De Jonnés,† "the fecundity of the people is restrained within the strictest limits."

"The rate of increase of the French population," says Mill, "is the slowest in Europe;‡ the number of births not increasing at all, while the proportion of births to the population is considerably diminishing."§

We have seen, moreover, that in France the actual ratio of living births is constantly and rapidly diminishing, while the still-births, actual and proportional, are as fast increasing; that the premature births progress in similar ratio, and by deduction and actual statistics, the criminal abortions; and that these facts obtain not merely in the metropolis, but throughout the country.

What are the causes of these remarkable facts, need it now be asked? Let all allowances be made for certain conjugal habits extensively existing among the French, and by no means rarely imitated in this country, but the proportionate decrease of living births is too enormous, the actual and proportionate increase of premature and still-births is too frightful to be wholly explained thus, or as West,‖ Husson,¶ and De Jonnés** have thought, to be attributed to a mere progressive lack of fecundity. Reason and the evidence alleged compel us to believe that in great measure they are owing to criminal abortion.

Political economists allow the facts in France to be as we have stated. Their interpretation of the causes, unwilling as they would be to confess its ultimate bearing, we now compel to serve as evidence.

---

* Report of 1849. † Loc. cit., p. 195. ‡ Loc. cit., i. p. 344. § Ibid., p. 343.

‖ Med. Times and Gazette, June 1856, p. 611.

¶ Les Consummations de Paris. ** Loc. cit.

"They depend," according to one writer,* "either on physical agents, especially climate, or on the degree of civilization of a people, their domestic and social habits." "In France the climate is favorable to an increase of population, and this obstacle, this restraint, is found in its advanced civilization."†

"This diminution of births," says Legoyt,‡ "in the presence of a constant increase of the general population and of marriages, can be attributed to nothing else than wise and increased foresight on the part of the parent."

"The French peasant is no simple countryman, no downright 'Paysan du Danube;' both in fact and in fiction he is now 'le rusé paysan.' That is the stage which he has reached in the progressive development which *the constitution of things has imposed on human intelligence and human emancipation*."§

"These facts are only to be accounted for in two ways. Either the whole number of births which nature admits of, and which happen in some circumstances, do not take place, or if they do, a large proportion of those who are born, die. The retardation of increase results either from mortality or prudence; from Mr. Malthus's positive, or from his preventive check; and one or the other of these must and does exist, and very powerfully too, in all old societies. Wherever population is not kept down by the prudence of individuals or of the State, it is kept down by starvation or disease."||

But on the other hand, it has been forgotten that the alternative supposed does not exist in the case we have instanced. Marriages in France, unlike some other continental States, are continually increasing, and starvation and disease are yearly being shorn of their power. The authors quoted are therefore forced to a single position; that the lessening of births can only be owing to "prudence" on the part of the community.

Moreover, it is allowed by Mill and by Malthus himself,¶ that so much of the decrease as cannot thus be explained, must be attributed to influences generally prevalent in Europe in earlier ages, and in Asia to the present time. "Throughout Europe these causes have much diminished, but they have nowhere ceased to exist."** Several of them have been named by the authority now quoted. Another, and greater than them all, he leaves unspoken; we are compelled to supply for him the omission.

The practice of destroying the fœtus in utero, to say nothing of infanticide, history declares to have obtained among all the earlier nations of the world, the Jews alone excepted, and to a very great extent. Aristotle

---

* De Jonnés, loc. cit., p. 194. † Ibid., p. 195. ‡ Journal des Economistes, 1847.

§ Mill, loc. cit., i. p. 336. The italics are my own. I shall hereafter refer back to this passage.

|| Ibid., i. p. 417. ¶ Essay on Population. ** Mill, loc. cit., i. p. 417.

defends it,* and Plato.† It is mentioned by Juvenal,‡ Ovid,§ Seneca, and Cicero, and is denounced by the earlier Christians.|| It was common in Europe through the middle ages, and still prevails among the Mohammedans,¶ Chinese,** Japanese,†† Hindoos,‡‡ and most of the nations of Africa and Polynesia,§§ to such an extent, that we may well doubt whether more have ever perished in those countries by plague, by famine, and the sword.

It is evident, therefore, that the actual and proportionate increase of still-births, and, by induction, setting aside all probable cases of infanticide, of abortion, and the comparative increase of a population reciprocally influence and govern each other so completely, that from the one it may in any given case be almost foreseen what the other must prove.

It is impossible that the results quoted from the Registry of New York, any more than those of France, even if so far, can in any great measure be owing to natural causes alone. They are wholly inexplicable on any principles, "which do not recognize an amount of guilt at which humanity shudders." In comparing that city with Paris, certain allowances must indeed be made; abroad, for the effects of wars and conscription, of despotism, and of migration outward; at home, for the effects of governmental laxity, and of migration inward. In both cities the amount of prostitution, an element not to be lost sight of, must be nearly the same; and in both, under the steady progress of science, medical and hygienic, the ratio of fœtal mortality, unless induced by criminal causes, may year by year be supposed to have been steadily diminished.

We have seen that in New York, in the absence of all influences that tend to keep down population in foreign countries, old and crowded, and under the yoke of despotism, the effects, attributable elsewhere to these causes, exist, and to a greater degree than in any other country;

That the ratio of fœtal deaths to the population had swelled from 1 in 1633, in 1805, to 1 in 340, in 1849; while in France, at a later period, 1851, they were only about 1 in 1000;

That the actual number of fœtal deaths in seven years, from 1850 to 1857, had very nearly doubled;

---

* Travels of Anacharsis, v. p. 270. † Ibid., iv. p. 342. ‡ Satires, vi., v. 592.

§ Amor., lib. 2.; Heroïdes, epist. 2. || REEVE's Apologies.

¶ BLAQUIERE, Letters from the Med., pp. 90, 184; SLADE, Records of Travels, ii. p. 162.

** BARROW, Travels in China, p. 113; DE PAUW, Philosoph. Dissert; MEDHURST, China, &c., p. 45; SMITH, Exploratory Visit, &c., i. p. 53.

†† GOLOWNIN, Memoirs of a Captivity, iii. p. 222.

‡‡ MOOR, Hindoo Infanticide, p. 63; BUCHANAN, Christian Researches in Asia, p. 49; WARD, View of the History, &c., of the Hindoos, p. 393.

§§ For a long list of authorities on these points, see BECK, ii. p. 389, et seq.

That the fœtal deaths, as compared with the total of births, elsewhere in cases of illegitimacy, where the results are the very worst, and where crime is confessed to have produced them, being 1 in 16·8,* had here, legitimate and natural, reached the frightful ratio of 1 in 8·1;

That the fœtal deaths, as compared with the total mortality, had increased from 1 in 37, in 1805, to 1 in 13, in 1855;

That the reported early abortions, of which the greater number of course escape registry, bear the ratio to the living births of 1 in 40·4, while elsewhere they are only 1 in 78·5;

And finally, that early abortions, bearing the proportion to the still-births at the full time of 1 in 10·2, in 1846, had increased to 1 in 4·02, in 1856.

It must be borne in mind that these statistics are positive, proving the existence of a certain number of pregnancies abruptly terminated. They cannot therefore be controverted by any argument regarding means for the prevention of pregnancy, no matter to what extent these may be used. Nor should it be forgotten that for every registered premature birth or abortion, innumerable ones occur that are never recorded.

Almost doubling therefore, as does New York, the worst of those fearful ratios of fœtal mortality existing in Europe, it is not strange that our metropolis has been held up, even by a Parisian, to the execration of the world. "On le voit (l'avortement)" says Tardieu, "en Amérique, dans une grande cité comme New York, constituer une industrie véritable et non poursuivie."

In this description of New York, we have that of the country.† The relative annual increase of the population existing throughout America, depending as this does chiefly on immigration, must not mislead us. The ratio of fœtal death in the metropolis surpasses what has ever been dreamed to obtain even in old countries, where innumerable more legitimate causes for it might be thought to exist. At Boston, which for morals is allowed to compare favorably with any city of its size in the Union, "undoubtedly more than a hundred still-births yearly escape being recorded, a large proportion of which, no doubt, result from criminal abortion."‡ And our

* Belgium.

† Local exceptions to this general rule will of course be found to exist, as is always the case with laws based on mere statistics, especially, as here, where reports to the registry are liable, for evident reasons, to be withheld. Thus it appears from Dr. Jewell's collections (this Journal, March, 1857, p. 277,) that the proportion of still-births in Philadelphia was, in 1856, only 1 in 913 to the total population, and 1 in 20·1 to the general mortality, against which evidence must be placed that which we subsequently furnish from Prof. Hodge.

‡ MSS. Letter from City Registrar, March 26, 1857

public prints, far and wide, even in the smaller towns and villages, constantly chronicle deaths from the commission of the crime.

In the State of Massachusetts at large, it has been found of late years that "the increase of the population, or the excess of the births over the deaths, has been *wholly* of those of *recent foreign origin;*"* this in 1850. In 1853 "it is evident that the births within the Commonwealth, with the usual increase, have resulted in favor of foreign parents in an increased ratio."† In other words, it is found that in so far as depends upon the American and native element, and in the absence of the existing immigration from abroad, the population of Massachusetts is stationary or decreasing.‡

"This result will doubtless surprise many, who will hardly think it possible. Is it general, or is it accidental? If it be general, how has it happened? What causes have been in operation to produce it? How is it to be accounted for?"§ These questions have hitherto been unanswered. We shall find, however, their solution only too easy.

Amid such general thrift and wealth, there has been every reason for the native, like the foreign population, to increase. The preventive check of the economists, though undoubtedly present, can have played but an inferior part, as we shall prove. Emigration westward, the only apparent positive check, though extensive, cannot wholly account for the result.

---

* CHICKERING, Comparative View of the Population of Boston, 1850. City Document, No. 60, p. 44.

† Twelfth Registration Report to the Legislature of Massachusetts, 1853, p. 116. The truth of this statement has been corroborated by Dr. Curtis, in his Report on the Census of Boston in 1855. City Document, 1856, p. 22. Also, Fifteenth State Registration Report, 1856, p. 179.

‡ "Had the rate of the annual increase of the numbers living under the age of five (3·13 per cent.) resulted entirely from the increase of births in a permanent population, the number of births of 1855 (in the districts where the ratio of the registered deaths to the population was greater than one to sixty-three, 166 of the 331 towns) would have been 24,457, instead of 23,481, the number registered. On the other hand, had the increase resulted wholly from migration, (the annual number of births in the permanent population being constant,) the number of births would have been only 22,956. The number of births registered is somewhat nearer the latter than the former of these two values.

"Assuming the correctness of the births, deaths, and population, in the selected districts, it appears that 35 per cent. of the increase of the population under the age of five was due to births in the permanent portion of the population, and 65 per cent. due to the movement of the migratory portion; also, that 38 per cent. of the increase of population at all ages was due to excess of births over deaths, leaving 62 per cent. to be accounted for by excess of immigration over emigration."—ELLIOTT, The Laws of Human Mortality in Massachusetts; Proceedings of Am. Assoc. for Adv. of Science, Montreal, 1857, p. 57.

§ CHICKERING, loc. cit., p. 49.

But statistics exist by whose light, if acknowledged, we may read this important problem.*

In 1850, the ratio of births to the population in Massachusetts, foreign and American combined, was 1 in 36,† and in 1855, 1 in 34;‡ a ratio much smaller than that obtaining in most countries of Europe, and about equaling that of France, which, in 1850, was 1 in 37.

In 1855, the ratio of still-births, at the full time and premature, as compared with the living births, was 1 to 15·5.§ In France it is 1 to 24; in Austria, 1 to 49.

In 1851, the ratio of fœtal deaths to the general mortality, was 1 to 13·3;‖ in 1855, 1 to 10·4.¶ In New York City, in 1856, it was 1 to 11· In any city we should expect to find the proportion much greater than in a State at large; we here find it less.

The ratio of premature births to those at the full time, during the period from 1850 to 1856, was 1 to 26·1.** In New York, in 1857, it was 1 to 40, and in good medical practice it is found, as we have seen, to be 1 in 78.

In comparing the recorded abortions and premature births in the City of New York, with the still-births there occurring at the full time, we found that the former had varied from 1 in 10, in 1838, to 1 in 4, in 1856.

In the State of Massachusetts it appears, that during the fourteen years and eight months preceding 1855, there were recorded 4570 still-births, and 11,716 premature births and abortions,†† the ratio being 1 abortion to ·3 still-birth; or, in other words, it would appear from the statistics quoted, that the comparative frequency of abortions in Massachusetts is *thirteen* times as great as in the worst statistics of the City of New York. We are willing, however, we rejoice, to modify this statement, as in the earliest of the years quoted, returns from the City of Boston seem to have

---

* The tables now presented, we have compiled from the fifteen published Registration Reports of the State of Massachusetts. Advance sheets of the Sixteenth Report have kindly been furnished me while this article is passing through the press, by the compiler, Dr. Josiah Curtis, of Boston. The premature births for 1856 and 1857 are not given in the reports for those years, so that I cannot extend my calculations beyond 1855. Deductions from the still-births at the full time, which are alone given in the years referred to, are of course useless for the present inquiry.

† Births, 27,664; population, 994,665.

‡ Births, 32,845; population, 1,132,369.

§ Total births at full time, 32,845; living births at full time, 32,120. Fœtal deaths, 2064; still, at full time, 725; premature, 1339.

‖ Total deaths, including 1462 fœtal, 19,461.

¶ Total deaths, including 2064 fœtal, 21,523.

** Births at full time, 154,245; premature, 5899.

†† Fourteenth Registration Report, 1855.

been imperfect or wanting,—and to confine our calculations to a more recent period.

From 1850 to 1855, the registration being much more accurate than before, and its results compiled with the greatest care, three years of the five by a noted statistician, Dr. Shurtleff, there were recorded in Massachusetts, 2976 still-births, and 5899 premature births and abortions,* the ratio being 1 abortion to ·5 still-birth; in other words, the frequency of abortions as compared with still-births at the full time, seems at least eight times as great in Massachusetts as in the worst statistics of the City of New York.†

It must not be forgotten that while nearly every still-birth at the full time is necessarily recorded, there must be but very few registrations of the premature births and abortions actually occurring; that though the contrary seems here the case, such occurrences are generally, as they would be supposed, far more frequent in crowded cities than in country districts, or in a State at large; and that however great may be the influence of the prevention of pregnancy in repressing a population, these constitute proofs of pregnancies actually occurring, and frequently criminally terminated.

Few persons could have believed possible the existence of such frightful statistics, the result toward which they must be confessed inevitably to tend, or the dread cause from which they spring. Either these statistics must be thrown aside as utterly erroneous and worthless, or they must be accepted with their conclusions. We would gladly do the former, but they present too many constant quantities, in other respects,‡ for this to be allowed. Our own calculations have been made with care, and we have presented the elements on which they rest. In asserting the results, at once so awful and astounding, we desire to fix upon them the attention and scrutiny of the world.

---

* Fourteenth Registration Report, 1855.

† In the above remarks we must not be misunderstood. We believe Massachusetts no worse with regard to abortion than many other portions of the country, but that its registration is conducted with greater care. From the statistics given it may easily be surmised what the amount of this crime *must be elsewhere.* It is necessarily of infinitely more common occurrence than infanticide, the murder of children after birth, for proof of the frequency of which, at the present moment, in Great Britain, we refer to Dr. Burke Ryan's Fothergillian Essay on the subject in the London Sanitary Review for last July, and to the London Lancet of corresponding date.

‡ As, for instance, in the regularly progressive series of deaths and births, as compared with the population; constant also as compared with each other:—Population of Massachusetts: by census of 1850, 994,665; 1855, 1,132,369. Deaths: 1851, 18,934; 1852, 18,482; 1853, 20,301; 1854, 21,414; 1855, 20,798. Births: 1851, 28,681; 1852, 29,802; 1853, 30,920; 1854, 31,997; 1855, 32,845.

We have seen that the increase of the population of Massachusetts by living births, is almost exclusively among its resident foreigners, Catholics, the rules of whose church will hereafter be shown to exercise an important influence in preventing the destruction of fœtal life. The conclusion cannot therefore well be avoided, that in these facts there exists the evident relation here intimated, of effects to cause.

With some certainty, then, even though statistics are as yet so imperfect, can we assert this conclusion, that frightful as is the extent to which the crime of abortion is perpetrated abroad, there is reason to believe that it prevails to an equal, if not even greater extent at home.

The frequency of arrests or trials for abortion afford, save indirectly, no criterion of the actual frequency of the crime. Our laws on this subject are at present so easily evaded, that officers of justice find it useless to trouble themselves with its prosecution; it is indeed "non poursuivie." During the eight years from 1849 to 1858, no report for 1853 being rendered by the Attorney-General, there were 32 trials for abortion in Massachusetts; and in these there was not a single conviction.* An estimate that for every arrest for this crime, a thousand instances of its commission escape the vigilance or at least the hand of the law, would probably be within the truth. That such is the fact, is shown by the statistics of France, where, from 1846 to 1850, out of 188 cases that came to the knowledge of the police, for lack of decisive evidence, only 22 went to trial,† or about one-ninth of those legally investigated. From 1826 to 1853, there were in France 183 trials for abortion. At the above ratio this will give about 1700 cases judicially examined, a yearly average throughout the empire of between 50 and 60. Comparing this fact with the statistics of the Morgue already given, and with those of the actual decrease of the rate of increase of the population in Paris and in New York, and the increase of premature births, our statement of its frequency will not seem exaggerated.

The number and success of professed abortionists is notorious. If arrested, they are always ready with bribes or abundant bail. Hardly a newspaper throughout the land that does not contain their open and pointed advertisements, or a drug-store whose shelves are not crowded with their nostrums, publicly and unblushingly displayed: the supply of an article presupposes its demand. From these facts we may fairly estimate the extent of their nefarious traffic.

That families are seldom now found of the size formerly common, is also

* Reports of Attorney-General of Massachusetts, from 1849 to 1858. State Documents.

† Comptes Rendus Annuels de la Justice Criminelle.

a matter of general remark. It were foolish to attempt to explain this by supposing that the present is an age of more moderate desire, or less unbridled lust; there is too much collateral proof against any such plea. Nor is it reasonable to think that women are generally becoming less productive of offspring than formerly, from any natural cause; or that the mass of our population, whatever the exceptions, are already so far advanced in knowledge, physiological or mechanical, or in practice, as in most cases to be able to regulate impregnation at will. The number of pregnancies must be nearly as abundant as ever; who can doubt what becomes of the offspring? We deny, simply and decidedly, the statement of some writers, that of every seven pregnancies, at least one always *naturally* terminates in abortion; this is uncorroborated by any reliable evidence, and is without doubt untrue.

Of the experience of physicians, there can be but one opinion. If each man of the profession were honestly to investigate this matter, and as honestly to avow the result, the mass of evidence would be overwhelming. This statement is in nowise invalidated by the experience alleged by many, especially among older practitioners; their evidence, based chiefly on lack of inquisition, or on the acknowledged less prevalence of the crime in former years, is merely negative, and as such only to be valued.

"We blush," says Prof. Hodge, "while we acknowledge the fact, that in this city, (Philadelphia,) where literature, science, morality, and Christianity are supposed to have so much influence; where all the domestic and social virtues are reported as being in full and delightful exercise, even here" it prevails.*

Dr. Blatchford, of Troy, N. Y., writes me thus: "A crime which forty years ago, when I was a young practitioner, was of rare and secret occurrence, has become frequent and bold." But why multiply instances of what must be the almost universal experience?

Applications for abortion are in many neighborhoods of constant occurrence, by no means among the poorer classes alone; and few women, unless also convinced by their physician of the enormity and guilt of that they intend, are deterred by his refusal from going elsewhere for aid, or from inducing abortion upon themselves.

But far greater proof than this we all possess, or can, if we desire. In but few of the abortions criminally induced is an application ever made to the physician in regular standing. He is oftener called upon after the crime has already been committed, to treat its acute and immediate effects. If he choose to take for granted in every case, that it has occurred from a perfectly natural cause, even where attending circumstances clearly point

* Introductory Lecture, 1854, p. 17.

to the contrary, or to ask no questions, or to shut his eyes and his ears to evident and patent facts, he can of course do so, and perhaps persuade himself that the crime is rare; but if he reflect that upon himself more than on clergyman or legislator, often rests the standard of public morals, and act accordingly, he may arrive at a different result.

But this is not only the fact in acute cases of abortion. The same statement holds true, perhaps even to a greater extent, with regard to chronic obstetric disease. It is now acknowledged that much of this is really the consequence of past difficult or abnormal labors; that the more complicated or improperly interfered with the labor has been, the more certain are unfortunate sequelæ; and that the earlier in pregnancy its occurrence, the greater, as a general rule, the danger, not merely to the mother's life, but to her subsequent health. In the treatment of these results, even more marked perhaps at a late period than earlier, the dependence of effect on cause, and their evident connection, can often be learned by a faithful inquirer, and in no small proportion of cases does the history go back without turn or the shadow of a doubt, to an induction of criminal abortion.

As a mere matter of individual experience, and from a practice by no means exceptional, the writer some time since reported no less than fifteen such cases as occurring to himself within hardly six months; and of these, all without exception were married and respectable women,* many of them of wealth and high social standing; and subsequently he was able, in consultation, to point out similar cases in the practice of gentlemen who, at that time, had denied the legitimacy of his conclusions. This experience must be a common one, only some lack the courage, as others lack the will, to investigate the matter; should they do so, they can come but to one result.

The frequency of maternal deaths, confessedly from criminal abortion, as gathered from published statements and mortuary reports, is also an item of importance in our summary of evidence. It is probable that in but few of the fatal cases really occurring, is foul play ever thought of, especially if the standing of the victim, and her previous history, have been such as to prevent or disarm suspicion; and on the other hand, while immediate death is undoubtedly a frequent result of induced abortion, it is, in proportion to the cases of its later occurrence, or of confirmed and chronic ill health, comparatively rare. From which it must be granted, that for every case thus made known, very many others must necessarily exist.

---

* Report to Suffolk Dist. Med. Society, May, 1857; New York Med. Gazette, July, 1857, p. 390; N. H. Journal of Medicine, July, 1857, p. 211.

We are compelled, from the preceding considerations, to acknowledge not merely that criminal abortion is of alarming frequency among us, but that its frequency is rapidly increasing; this having been made apparent by each link in the chain of evidence that has been presented. Every effort that might possibly check this flood of guilt will, if delayed, have so much the more to accomplish. The crime is fast becoming, if it has not already become, an established custom, less honored in the breach than in the observance.

What are the causes of this general turpitude?

They also may be classified—

I.—The low *morale* of the community as regards the guilt of the crime.
II.—The ease with which its character, in individual cases, may be concealed.
III.—The unwillingness of its victims to give testimony that would also criminate themselves.
IV.—The possibility of their inducing abortion upon themselves without aid.
V.—The ease with which the laws, as at present standing, may be evaded.
VI.—The lack or inefficacy of judicial preventives; such as statutes for registration, and those against concealment of birth and secret burials.
VII.—The prevalent ignorance of the true principles of its jurisprudence in both government officials and medical witnesses.
VIII.—Social extravagance and dissipation.
IX.—The doctrines of political economists.
X.—The fear of child-bed.

That public opinion should at present attach so little importance to the value of fœtal life, has already been shown to be owing in great measure to ignorance respecting its actual existence in the earlier months of pregnancy. Two other, and no less general, physiological errors prevail, extensively inculcated by popular authors and lecturers for their own sinister purposes.

One of these is the doctrine that it is detrimental to a woman's health to bear children beyond a certain number, or oftener than at certain stated periods, and that any number of abortions are not merely excusable, as preventives, but advisable; it being entirely forgotten that the frequency of connection may be kept within bounds, and the times of its occurrence regulated, by those who are not willing to hazard its consequences; that if women will, to escape trouble or for fashion's sake, forego the duty and privilege of nursing, a law entailed upon them by nature, and seldom neglected without disastrous results to their own constitutions, they must expect more frequent impregnation; that the habit of aborting is generally attended with the habit of more readily conceiving; and that abortions, accidental, and still more if induced, are generally attended by the loss of subsequent health, if not of life.

This error is one which would justify abortion as necessary for the mother's own good; a selfish plea. The other is based on a more generous motive. It is that the fewer one's children, the more healthy they are likely to be, and the more worth to society. It is, however, equally fallacious with the first, and is without foundation in fact. The Spartans and Romans, so confidently appealed to, gave birth probably to as many weakly children as do our own women; that they destroyed many for this reason in infancy, is notorious. The brawny Highlanders are not the only offspring of their parents; the others cannot endure the national processes of hardening by exposure and diet, and so die young from natural causes. But were this theory true even so far as it goes, the world, our own country, could ill spare its frailer children, who oftenest, perhaps, represent its intellect and its genius.

In asserting that the doctrines of the leading political economists for the last half century are accountable for much of the prevalence and increase of the crime, ignorant or careless as these writers all seem of the dire means that would be resorted to for the attainment of their ends, I have in no way exaggerated. Malthus remarks in his well-known Essay on Population, that "in the average state of a well-peopled territory, there cannot well be a worse sign than a large proportion of births, nor can there well be a better sign than a small proportion;"* and he endorses the assertion of Hume, subsequently proved false by Sadler,† that the permission of child-murder, by removing the fear of too numerous a family in case of marriage, tends to encourage this step, and thereby the increase of population; "the powerful yearnings of nature preventing parents from resorting to so cruel an expedient, except in extreme cases."‡

Sismondi§ and a host of others might also be quoted, but a few extracts from a later writer, standard in this country at present, and taught in our universities, till very lately in that at Cambridge, for instance, will suffice.

"We greatly deprecate," says Mill, "an increase of population, as rapid as the increase of production and accumulation."‖

---

* Loc. cit., p. 313.

† The Law of Population, 1830.

‡ HUME, Essays, vol. i. No. xi., p. 431.

§ Etudes sur l'Economie Politique; Nouveaux Principes d'Economie Politique.

‖ Loc. cit., ii. p. 253.

"There is room in the world, no doubt, and even in old countries, for an immense increase of population. But although it may be innocuous, I confess I see very little reason for desiring it."

"I sincerely hope, for the sake of posterity, that they will be content to be stationary long before necessity compels them to it."*

"If the opinion were once generally established among the laboring class, that their welfare required a due regulation of the numbers of their families, only those would exempt themselves from it who were in the habit of making light of social obligations generally."† "The principles contended for include not only the laboring classes, but all persons, except the few who being able to give their offspring the means of independent support during the whole of life, do not leave them to swell the competition for employment."‡

"When persons are once married, the idea never seems to enter any one's mind, that having or not having a family, or the number of which it shall consist, is at all amenable to their own control. One would imagine that it was really, as the common phrases have it, God's will and not their own, which decided the number of their offspring."§

"In a place where there is no room left for new establishments," says Sismondi, entirely ignoring the escapes offered by emigration and the increased importation of food, "if a man has eight children, he should believe that unless six of them die in infancy, these, and three of his own contemporaries of each sex, will be compelled to abstain from marriage in consequence of his own imprudence."||

The direct result of remarks like these last, so pointed and plainly to be understood, is seen in the statistics I have so largely given. Would mankind, in following such advice, merely resort to greater abstinence before their means allow the expense of children, and to greater prudence after that period, no fault could be found; but when we discover criminal abortion thus justified and almost legitimated, we may well oppose to such doctrine the words of the indeed admirable Percival, "To extinguish the first spark of life is a crime of the same nature, both against our Maker and society, as to destroy an infant, a child, or a man."¶

Fear of child-bed, in patients pregnant for the first time, or who had suffered or risked much in previous labors, might formerly have been allowed some weight in excuse, but none at all in these days of anæsthesia. It has been urged, and not so absurdly as would at first sight appear, that

---

* Ibid., i. p. 451. An opinion to the same effect, italicized, has already been quoted.

† Ibid., ii. pp. 316, 317.

‡ Ibid., i. p. 452, foot-note.

§ Ibid., i. p. 447.

|| Nouveaux Principes, &c., liv. vii. ch. 5.

¶ Medical Ethics, p. 79.

the present possibilities of painless and so much safer delivery, by changing thus completely the primal curse, from anguish to a state frequently of positive pleasure, remove a drawback of actual advantage, and by offering too many inducements for pregnancy, tend to keep women in that state the greater part of their menstrual lives.

The consideration in detail of the various other causes to which I have alluded as accounting for the prevalence of abortion, together with that of the many special reasons offered in individual cases by way of excuse, I postpone for the present; merely premising that where ignorance is so evidently and so extensively its foundation, those who, possessing, yet withhold the knowledge which by any chance or in any way would tend to prevent it, themselves become, directly, and in a moral sense, responsibly accountable for the crime.

[TO BE CONTINUED.]

